# Mixed-methods formative evaluation of implementing an adapted suicide prevention treatment: Dialectical Behavior Therapy Skills Groups in the Veterans Health Administration

**DOI:** 10.3389/fpsyt.2024.1495102

**Published:** 2024-11-25

**Authors:** Suzanne E. Decker, Aimee Kroll-Desrosiers, Kristin Mattocks, Frances M. Aunon, Elizabeth Galliford, Neal Doran, Scarlett Baird, Jennifer K. Rielage, Josephine Ridley, Jenny Bannister, Thorayya S. Giovannelli, Sara J. Landes, Marianne Goodman, Lorrie Walker, Eric DeRycke, Chris Shriver, Ethan Spana, Mark Honsberger, Hannah Brown, Stacey Demirelli, Elena Shest, Steve Martino

**Affiliations:** ^1^ VA Connecticut Healthcare System, West Haven, CT, United States; ^2^ Department of Psychiatry, Yale School of Medicine, New Haven, CT, United States; ^3^ VA Central Western Massachusetts Health Care System, Leeds, MA, United States; ^4^ Department of Population and Quantitative Health Sciences, University of Massachusetts T.H. Chan School of Medicine, Worcester, MA, United States; ^5^ VA San Diego Healthcare System, San Diego, CA, United States; ^6^ Department of Psychiatry, University of California San Diego School of Medicine, San Diego, CA, United States; ^7^ VA New Mexico Healthcare System, Albuquerque, NM, United States; ^8^ Department of Psychiatry and Behavioral Sciences, University of New Mexico School of Medicine, Albuquerque, NM, United States; ^9^ VA Northeast Ohio Healthcare System, Cleveland, OH, United States; ^10^ Department of Psychological Sciences, Case Western Reserve University, Cleveland, OH, United States; ^11^ James A. Haley Veterans Hospital, Tampa, FL, United States; ^12^ Behavioral Health Quality Enrichment Research Initiative (QUERI), Central Arkansas Veterans Healthcare System, North Little Rock, AR, United States; ^13^ Department of Psychiatry, University of Arkansas for Medical Sciences, Little Rock, AR, United States; ^14^ Mental Illness Research Education and Clinical Center (MIRECC), Veterans Integrated Service Network (VISN) 2, James J. Peters Department of Veterans Affairs Medical Center, The Bronx, NY, United States; ^15^ Department of Psychiatry, Icahn School of Medicine at Mount Sinai, New York, NY, United States

**Keywords:** suicide prevention, Veterans Health Administration, i-PARIHS, Dialectical Behavior Therapy, psychotherapy, implementation, mixed methods, emotion dysregulation

## Abstract

**Background:**

Preventing veteran suicide requires addressing mechanisms driving suicidal behavior, such as emotion dysregulation. Dialectical Behavior Therapy Skills Groups (DBT-SG) are well established for reducing emotion dysregulation, improving coping skills, and in some studies, reducing suicide attempt, but will require implementation support to deliver DBT-SG and to test its effectiveness within the Veterans Health Administration (VHA).

**Methods:**

We conducted a mixed-method developmental formative evaluation of DBT-SG at four VHA medical centers, guided by the Integrated Promoting Action on Research Implementation in Health Services (i-PARIHS) framework, as part of a hybrid effectiveness-implementation trial (Clinical trials ID, NCT05000749).

**Results:**

Quantitative Organizational Reasons for Change Assessment data (*n* = 30 VHA staff) and qualitative data (*n* = 35 VHA staff) were merged, compared, and triangulated. Quantitative and qualitative data largely converged, showing favorable views of evidence supporting DBT-SG and strong enthusiasm for its potential to reduce veteran suicide attempt. Staff noted DBT-SG’s broad applicability to veterans. Staff were less optimistic about the inner context supporting DBT-SG implementation, commenting on how limited staffing could be a barrier despite leadership wanting to support suicide prevention.

**Conclusions:**

Implementation barriers to DBT-SG at VHA include limited staffing, despite staff enthusiasm. The next phase of this project will evaluate DBT-SG effectiveness in a randomized controlled trial.

**Clinical trials registration:**

https://clinicaltrials.gov/study/NCT05000749, identifier NCT05000749.

## Introduction

In the United States, veterans of the Armed Forces die by suicide at an age- and sex-adjusted rate 71.8% greater than non-veteran adults (1). Veteran suicide prevention is a top priority of the United States government including the Department of Defense, Veterans Affairs, and Health and Human Services (2). The Department of Veterans Affairs has a multicomponent strategy to prevent veteran suicide, including promoting effective treatments for veterans identified to be at risk (2, 3). Many different mental health disorders are associated with increased suicide risk among veterans (4), suggesting a transdiagnostic approach focusing on cross-cutting risk factors is needed. Emotion dysregulation, or difficulties regulating emotions, occurs across mental health diagnoses and has been identified as a transdiagnostic treatment target (5). Emotion dysregulation is associated with suicide attempt frequency among those at risk for suicide (6), even when controlling for other risk factors (7, 8). Skills training in emotion regulation is a critical component of effective treatments for reducing suicide attempt (9).

Dialectical Behavior Therapy (DBT) is an intervention focusing on emotion regulation skills training (10) that is well supported for reducing self-directed violence, including suicide attempt (11). While DBT was originally studied in individuals with borderline personality disorder (BPD), a disorder characterized by high levels of emotion dysregulation (12), DBT has increasingly been utilized with a broad range of individuals at elevated risk for suicidal behavior (e.g., 13, 14), including veterans (15). Comprehensive DBT is a multi-component intervention including individual therapy, a skills training group providing instruction in emotion regulation and other skills, phone coaching between sessions, and a therapist consultation team (10). In 2020, VHA launched the “Suicide Prevention 2.0” program, with both community- and clinical-based intervention strategies (2). Since 2021, the clinical arm of this initiative, SP 2.0 Clinical Telehealth, has partnered with the VHA regional Clinical Resource Hubs, which provide telehealth to veterans, to provide evidence-based treatment for suicide prevention to veterans with a history of suicidal self-directed violence. Comprehensive DBT is one of the four treatments offered, specifically for veterans who are also living with BPD (16).

DBT is a complex intervention that requires some unique treatment elements, such as two group leaders for each skills group session, weekly consultation team for therapists, and access to therapists between sessions (17). Unpaid or unique elements of DBT may conflict with existing healthcare system structures or clinicians’ schedules (17). Implementation barriers to comprehensive DBT in public health settings are well documented in qualitative or mixed-method studies, and include concern about the fit of the intervention with the clinic’s pre-existing practices or structure (18), limited staff time or competing productivity demands (18–21), insufficient administrative support (19), absence of or limited administrator buy-in (19, 20), low resource availability (20, 22), challenges related to phone coaching (18, 21), the perceived difficulty of implementing DBT (22), and turnover of trained practitioners (18–20, 22). Concerns about the degree of fit between DBT and the healthcare system’s current practices may contribute to only some modes of DBT being implemented (17, 23), or even to healthcare settings stopping DBT implementation (23).

Newer data suggest the DBT skills group, supported by therapist consultation team, may be the treatment’s active ingredient. In adults with BPD at high risk for suicide, DBT skills group with consultation team (DBT-SG) and rigorous risk management and individual manualized case management services reduced suicide attempt and ideation with the same efficacy as comprehensive DBT (24). Similarly, among individuals with BPD, DBT-SG resulted in reduced suicidal and nonsuicidal self-injurious behavior and reduced affect instability (24). Additionally, DBT-SG increased coping or distress tolerance skills (25, 26) among BPD and transdiagnostic samples. DBT-SG has been applied to a wide variety of diagnoses, suggesting its utility well beyond the BPD diagnosis (27). While the DBT-SG innovation has high potential to reduce suicide attempt and emotion dysregulation across a range of mental health disorders, its implementation determinants have not been prospectively studied.

Successful implementation of an innovation requires understanding context-specific factors (17), including the availability of behavioral health providers (28) and their workload demands (29). DBT-SG innovation features, such as the therapist consultation team, may also affect implementation (17, 28) or require specific implementation strategies (30). To understand implementation determinants of DBT-SG among veterans at high risk for suicide attempt, we conducted a mixed-method formative evaluation, guided by the Integrated Promoting Action on Research Implementation in Health Services (i-PARIHS) framework (31), to identify how characteristics of the DBT-SG innovation, inner and outer context, and recipients may impede or assist DBT-SG implementation. This formative evaluation was conducted at outpatient mental health clinics across and within four VHA medical centers as a prelude to a multi-site effectiveness trial.

## Materials and methods

### Study design

#### Study setting and context

VHA is a national healthcare organization serving United States Armed Services veterans and their dependents. The overall study is an ongoing hybrid type 1 effectiveness-implementation (32, 33) multi-site randomized clinical trial examining DBT-SG plus VHA treatment as usual versus VHA treatment as usual to reduce suicide attempt among veterans with emotion dysregulation who are at high-risk for suicide attempt. The trial is being conducted at four VHA medical centers in different geographic regions of the United States. The trial was reviewed and approved by the Central Institutional Review Board of the VHA, and written informed consent was waived for the collection of these survey and interview data.

#### Implementation framework and design

The formative evaluation is guided by the i-PARIHS framework (31). Successful implementation is a function of the interplay between the innovation (its evidence, usability, and fit with local practice and priorities), its recipients (clients who receive the intervention and healthcare staff who implement it, and their skills, resources, and motivations), and the context (local, organizational, and external leadership support, organizational priorities, mandates, and incentives). The i-PARIHS framework was chosen for its emphasis on context, which has been identified as particularly important in DBT implementation (30).

The formative evaluation uses a longitudinal convergent parallel mixed-method design with concurrent, but separate, quantitative and qualitative data collection, thus giving equal weight to each type of data (QUAN + QUAL (34);. Data collection included (1) a quantitative survey about organizational readiness to change and brief demographic survey, and (2) qualitative semi-structured telephone interviews, each described in detail below. These data are from a developmental formative evaluation (35) conducted prior to clinical trial initiation.

### Participants

VHA staff who had agreed to implement DBT-SG (study therapists) or would be affected by the implementation of DBT-SG (local clinicians who might refer veterans to DBT-SG; local suicide prevention coordinators; local clinical leadership; national VHA leadership) were recruited using email and invited to participate. Inclusion criteria were being a VHA staff member in one of the above roles; the exclusion criteria were being unable to read English or communicate in written and spoken English. Of the 72 staff approached, the total sample included 41 VHA staff (*n*=24 who provided both qualitative and quantitative data*, n*=6 who provided only quantitative data, and *n*=11 who provided only qualitative data).

### Procedure

#### Quantitative data

VHA medical center staff (excluding national VHA leadership given unfamiliarity with site-specific issues) were invited via email to enter survey responses directly into REDCap, a secure, web-based data capture tool (36). Measures included the Organizational Readiness for Change (ORCA) instrument, which demonstrates good internal consistency and factor structure (37), and a brief demographic instrument. The ORCA instrument is comprised of 77 items that are grouped into subscales corresponding to the main areas of the initial PARIHS framework. Participants were asked to respond only to items involving their perceptions of DBT-SG evidence (the nature and strength of the evidence and its potential for implementation; four subscales) and implementation context (the environment or setting in which the proposed change is to be implemented; six subscales), as items related to facilitation presume experience with DBT-SG’s actual implementation, which had not yet started during the pretrial developmental formative evaluation. Respondents rated each item from 1 to 5 (1=Strongly Disagree, 2=Disagree, 3=Neither Agree nor Disagree, 4=Agree, 5=Strongly Agree).

#### Qualitative data

VHA staff, including mental health VHA national operational and local medical center leaders, were invited to participate in an audio recorded individual telephone or videoconference interview of about 30 minutes’ duration. Interviews were semi-structured and guided by interview guides for each participant type (study therapist, referring clinician, suicide prevention coordinator, local mental health leadership, VHA national mental health leadership; guides available from first author upon request). Interview items explored respondents’ perceptions about inner context (e.g., local organizational factors), outer context (e.g., policy drivers and priorities), the innovation (e.g., DBT-SG evidence-base and usability), recipient characteristics (e.g., goals, skills and knowledge, resources, and support), and potential facilitation (e.g., roles and services that might support DBT-SG adoption). A sample size up to sixty participants was sought to identify site-specific implementation determinants, consistent with a similarly large sample in another multisite VHA trial (38). Interviews were conducted by an expert qualitative methodologist (redacted) and her team.

### Analyses

#### Quantitative

We calculated descriptive statistics for each sample: 1) VHA staff who completed the ORCA; 2) VHA staff who completed a qualitative interview. We described each of these samples by gender, age, race, and other sample specific characteristics. Frequency and percent or mean and standard deviation (*SD*) were calculated as appropriate.

Next, we calculated descriptive statistics for overall and site-specific ORCA scores. We began by examining descriptive statistics for each ORCA item and then 1) calculating overall ORCA scores, 2) scores for the evidence and context domains, and 3) scores for the individual subscales within the evidence and context domains (*n*=4 and *n*=6 subscales, respectively). Subscale scores were depicted graphically by site to compare responses between the four medical centers. Additionally, we examined subscales within the evidence and context domains for referring providers/study therapists (*n*=8) vs. others and separately for participants in leadership roles (*n*=9) vs. others. No statistical comparisons were conducted to compare sites given our relatively small sample size; however, we did examine statistical differences using student’s t-tests to compare referring providers/study therapists vs. others and those in leadership roles vs. others.

#### Qualitative

Qualitative data were evaluated using an established rapid content analysis method developed within VHA (39, 40) and used in prior hybrid effectiveness-implementation studies (41). Analyses occurred throughout data collection to allow for reduction of data and incorporation of new emerging categories or themes. At the time of each interview, the interviewer took notes in an electronic summary template document to summarize the interview content into domains based on the semi-structured interview questions (39). Emerging categories were transferred into matrices, and matrix analysis methods were used to identify key themes across interviews. Matrices systematically noted the similarities, differences, and trends in responses across interviews, expediting synthesis and summary of findings (42). Analyses focused on identifying themes across respondents, with interpretation guided by the i-PARIHS framework. Cross-cutting themes became the framework for final analysis, which utilized a hybrid deductive and inductive analytic approach (43) in which established themes were evaluated against the data, while new themes were incorporated into the evolving coding scheme (44).

#### Triangulation of quantitative and qualitative data

Following the initial analyses of our data, our two types of data (quantitative and qualitative) were merged, compared, and triangulated. We integrated our findings to examine the extent to which our quantitative and qualitative results converged or diverged. Using a primary data analysis integration procedure, we identified common concepts and content areas across both sets of findings and synthesized our results.

## Results

### Sample description

#### Quantitative respondents

Thirty VA staff (response rate = 41.7%) across the four study sites completed the ORCA survey (**Table 1**). Respondents were majority female (77%), white (90%), an average age of 45 (+/- 9) years old, and all had an education level of a Master’s degree or higher.

**Table 1:** Characteristics of Study Participants, by Study (Quantitative/Qualitative) and Site.

Quantitative Data: VHA Staff (*n*=30)					
	Site A (*n*=7)	Site B (*n*=11)	Site C (*n*=7)	Site D (*n*=5)	Overall (*n*=30)
Age, mean (+/- SD)	47.7 +/- 11.6	45.7 +/- 7.1	45.9 +/- 9.8	40.2 +/- 9.5	45.3 +/- 9.2
Female gender, *n* (%)	5 (71.4)	8 (72.7)	5 (71.4)	5 (100.0)	23 (76.7)
Race*, *n* (%)						
White	7 (100.0)	10 (90.9)	5 (71.4)	5 (100.0)	27 (90.0)
Black	0 (0.0)	1 (9.1)	0 (0.0)	0 (0.0)	1 (3.3)
Other	0 (0.0)	0 (0.0)	1 (14.3)	0 (0.0)	1 (3.3)
Hispanic or Latino, *n* (%)	1 (14.3)	2 (18.2)	0 (0.0)	0 (0.0)	3 (10.0)
Field, *n* (%)						
Psychology	4 (57.1)	5 (45.4)	3 (42.9)	3 (60.0)	15 (50.0)
Psychiatry	1 (14.3)	1 (9.1)	1 (14.3)	0 (0.0)	3 (10.0)
Social Work	2 (28.6)	5 (45.4)	1 (14.3)	2 (40.0)	10 (33.3)
Counseling	0 (0.0)	0 (0.0)	1 (14.3)	0 (0.0)	1 (3.3)
Other	0 (0.0)	0 (0.0)	1 (14.3)	0 (0.0)	1 (3.3)
Doctoral degree, *n* (%)	5 (71.4)	6 (54.6)	5 (71.4)	3 (60.0)	19 (63.3)
Leadership Role, *n* (%)	2 (28.6)	4 (36.6)	2 (28.6)	1 (20.0)	9 (30.0)
Study Therapist or Referring Provider, *n* (%)	1 (14.3)	3 (27.3)	2 (28.6)	2 (40.0)	8 (26.7)
Qualitative Data: VHA Staff (*n*=32)						
	Site A (*n*=6)	Site B (*n*=9)	Site C (*n*=8)	Site D (*n*=9)	Overall (*n*=32)
Age, mean (+/- SD)	57.8 +/- 3.3	46.3 +/- 8.8	47.3 +/- 9.4	39.8 +/- 8.3	46.9 +/- 9.9
Female gender, *n* (%)	5 (83.3)	8 (88.9)	5 (62.5)	7 (77.8)	25 (78.1)
Race*, *n* (%)						
White	5 (83.3)	8 (88.9)	7 (87.5)	6 (66.7)	26 (81.3)
Black	1 (16.7)	1 (11.1)	0 (0.0)	0 (0.0)	2 (6.3)
Hispanic or Latino, *n* (%)	1 (16.7)	0 (0.0)	1 (12.5)	2 (22.2)	4 (12.5)
Field, *n* (%)						
Psychology	2 (33.3)	4 (44.4)	6 (75.0)	3 (33.3)	15 (46.9)
Psychiatry	1 (16.7)	0 (0.0)	1 (12.5)	2 (22.2)	4 (12.5)
Social Work	2 (33.3)	3 (33.3)	1 (12.5)	4 (44.4)	10 (31.2)
Other	1 (16.7)	2 (22.2)	0 (0.0)	0 (0.0)	3 (9.4)
Doctoral degree, *n* (%)	3 (50.0)	6 (66.7)	6 (75.0)	5 (55.6)	20 (62.5)

*No participants identified as Asian or Pacific Islander or American Indian or Alaska Native. Two participants from Site D and one participant from Site C did not report race or ethnicity. Columns may not sum to 100% due to missing data. SD, standard deviation. Demographic data for hospital and national leadership (*n* = 3) are not reported.

#### Qualitative respondents

Qualitative interviews (response rate = 44.4%) were conducted with 32 VHA staff and 3 members of VHA leadership (**Table 1**). Staff respondents were majority female (78%), white (81%), an average age of 47 (+/- 10) years old, and 97% had an education level of a Master’s degree or higher (**Table 1**). VHA leadership represented hospital and national level leaders (demographics not reported due to small sample size).

### Findings

#### Quantitative

**Figure 1:**
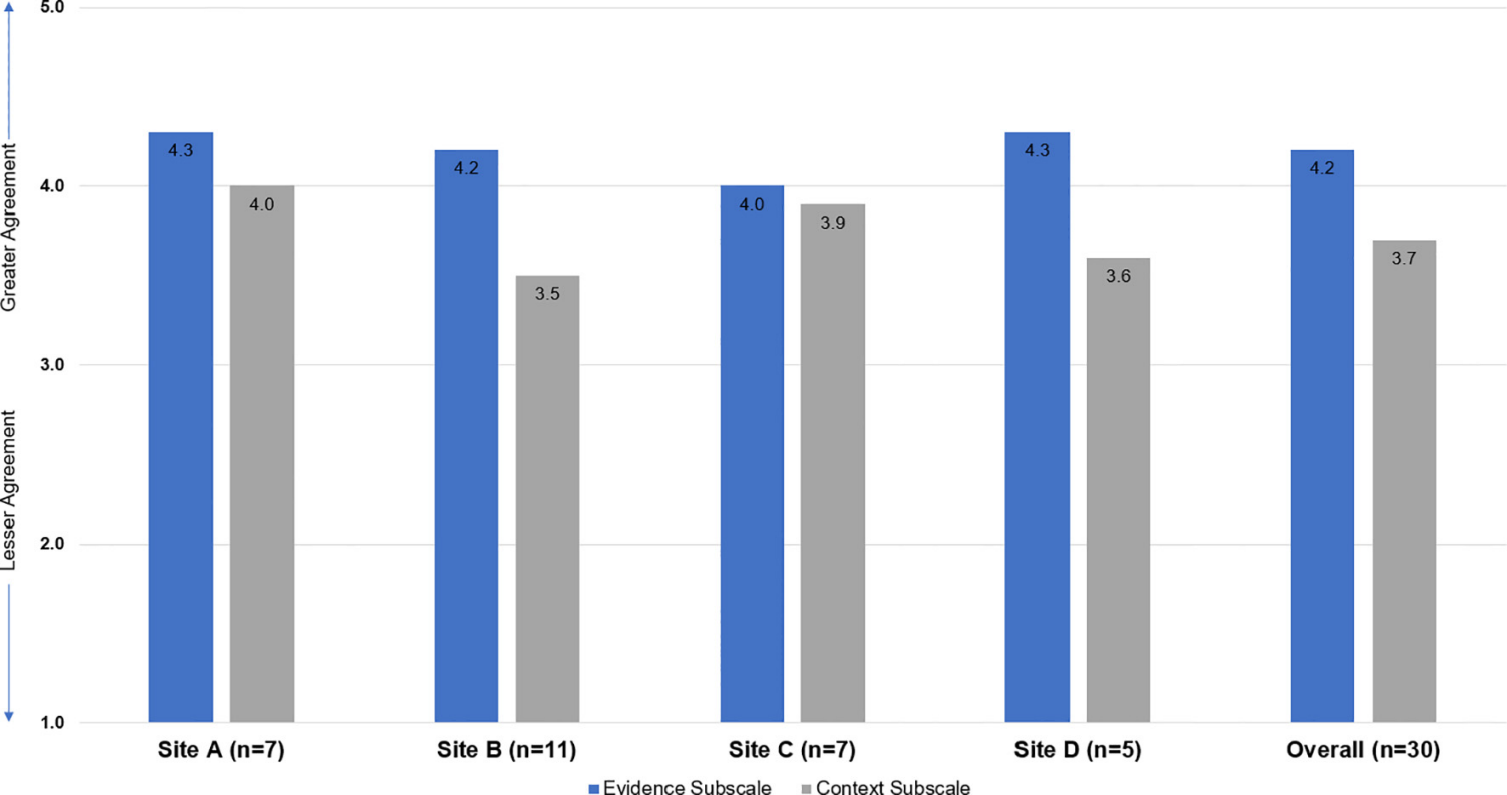
Mean Evidence and Context ORCA Subscale Scores - By Site and Overall. The Organizational Readiness to Change Assessment (ORCA; Helfrich, Sharp, & Sales, 2009) included items from the three domains of the original Promoting Action on Research in Health Services (PARIHS; Kitson, Harvey, & McCormack, 1998) assessing the strength of evidence for the innovation, quality of the context for implementation, and capacity to facilitate the implementation. This study used the ORCA evidence and context subscales. All quantitative items are scored as 1=Strongly Disagree | 2=Disagree | 3=Neither Agree nor Disagree | 4=Agree | 5=Strongly Agree.

Overall, respondents indicated agreement that the DBT-SG innovation is supported by evidence from RCTs, clinical experience, and patient preferences (mean ± *SD* scores across all sites: 4.1 ± 0.7, 4.3 ± 0.6, and 4.1 ± 0.5, respectively). However, respondents were in less agreement about support from the context including leadership culture (mean ± *SD*: 3.8 ± 1.0), leadership behavior (mean ± *SD*: 3.5 ± 1.1), feedback from leadership (mean ± *SD*: 3.5 ± 1.0), opinion leaders (mean ± *SD*: 3.8 ± 0.9), and general resources (mean ± *SD*: 3.3 ± 0.9) in providing a successful environment to implement DBT-SG. Despite these lower ratings on the context scale, staff culture remained high among respondents who indicated agreement that staff members have a sense of personal responsibility for improving patient care and outcomes (mean ± *SD*: 4.4 ± 0.7). There were minimal differences in ORCA ratings by site (**Table 2**; **Figure 1**).

**Table 2:** Mean ORCA Subscale Scores by Site and Overall (reported by VHA Staff, *n*=30).

	Site A (*n*=7)	Site B (*n*=11)	Site C (*n*=7)	Site D (*n*=5)	Overall (*n*=30)
	Mean ± Standard Deviation				
Evidence for the Innovation					
Randomized controlled trials	4.4 ± 0.6	4.0 ± 0.7	4.2 ± 0.7	4.0 ± 0.8	4.1 ± 0.7
Clinical experience	4.5 ± 0.4	4.3 ± 0.7	3.9 ± 0.7	4.5 ± 0.5	4.3 ± 0.6
Patient preference	4.3 ± 0.5	4.1 ± 0.6	4.0 ± 0.6	4.2 ± 0.3	4.1 ± 0.5
Context					
Leadership culture	3.9 ± 1.0	3.6 ± 1.1	4.0 ± 1.1	3.7 ± 0.7	3.8 ± 1.0
Staff culture	4.5 ± 0.4	4.2 ± 0.9	4.3 ± 0.6	4.6 ± 0.4	4.4 ± 0.7
Leadership behavior	3.9 ± 0.8	3.2 ± 1.2	4.0 ± 0.9	3.0 ± 1.0	3.5 ± 1.1
Feedback from leadership	3.7 ± 0.6	3.4 ± 1.2	4.0 ± 0.8	2.9 ± 0.8	3.5 ± 1.0
Opinion leaders	4.2 ± 0.8	3.5 ± 1.0	3.9 ± 0.6	3.5 ± 0.9	3.8 ± 0.9
Resources	3.7 ± 0.5	3.1 ± 1.1	3.2 ± 1.2	3.5 ± 0.5	3.3 ± 0.9

ORCA, Organizational Readiness for Change Assessment. Significance testing comparing sites was not conducted. ORCA items are scored from 1 (strongly disagree or very infrequently) to 5 (strongly agree or very frequently) with higher subscale scores showing greater agreement that DBT-SG implementation at VHA site is supported by the construct.

When we examined differences in ORCA scores by provider type, both for referring providers/study therapists and separately for leadership, we found that scores significantly varied for referring providers/study therapists vs. others (**Table 3A**) and leadership vs. others (**Table 3B**) for context items. With the exception of opinion leaders, referring providers/study therapists consistently rated context items lower than others (all *p*<0.05). With the exception of staff culture and opinion leaders, those in leadership roles consistently rated context items higher than others (all *p*<0.03).

**Table 3A:** Comparison of ORCA subscale scores by referring provider or study therapist designation (reported by VHA staff, *n*=30).

	Referring Provider or Study Therapist (*n*=30)		p-value
	Yes (*n*=8)	No (*n*=22)	
Evidence for the Innovation			
Randomized controlled trials	4.4 ± 0.6	4.1 ± 0.7	0.28
Clinical experience	4.6 ± 0.7	4.2 ± 0.6	0.16
Patient preference	4.3 ± 0.6	4.1 ± 0.5	0.51
Context			
Leadership culture	3.0 ± 1.1	4.1 ± 0.8	0.01
Staff culture	3.9 ± 0.9	4.5 ± 0.5	0.02
Leadership behavior	2.9 ± 0.7	3.8 ± 1.1	0.04
Feedback from leadership	2.8 ± 0.7	3.8 ± 1.0	0.02
Opinion leaders	3.3 ± 0.6	3.9 ± 0.9	0.06
Resources	2.5 ± 0.6	3.6 ± 0.8	0.001

**Table 3B:** Comparison of ORCA subscale scores by leadership* designation (reported by VHA staff, *n*=30).

	Leadership Role (*n*=30)		p-value
	Yes (*n*=9)	No (*n*=21)	
Evidence for the Innovation			
Randomized controlled trials	4.0 ± 0.4	4.2 ± 0.7	0.50
Clinical experience	4.1 ± 0.5	4.4 ± 0.7	0.32
Patient preference	4.0 ± 0.5	4.2 ± 0.6	0.37
Context			
Leadership culture	4.4 ± 0.5	3.1 ± 1.0	0.02
Staff culture	4.6 ± 0.5	4.3 ± 0.7	0.20
Leadership behavior	4.3 ± 0.7	3.2 ± 1.0	0.01
Feedback from leadership	4.3 ± 0.7	3.2 ± 0.9	0.002
Opinion leaders	4.2 ± 0.6	3.6 ± 0.9	0.06
Resources	4.0 ± 0.8	3.0 ± 0.8	0.01

*Leadership roles included hospital directors (n=1) and administrative leaders (e.g., Chief of Mental Health, clinical supervisors; n=8).

#### Qualitative

##### DBT-SG innovation

Providers across the four sites agreed that the evidence for DBT-SG was promising. They specifically identified the skills-based approach as valuable. The less resource-intensive DBT-SG intervention was lauded as being easier to implement. As one provider said:

*“Some DBT is better than no DBT, so even if you have a skills group alone without that individual component or that coaching component, I still think that would be really useful and beneficial for our high-risk veterans. I’m fully confident and hopeful that it will be effective.” – Participant 11*

Similarly, providers highlighted how a time-limited DBT-SG could provide foundational skills for veterans while fitting into the healthcare system’s model for shorter, evidence-based care approaches:

*“Our VA is trying very hard to streamline and use evidence-based short-term models and to deliver care in courses of treatment that are generally about 12 to 16 weeks. A 24-week skills group would be a great foundation for veterans to receive.” – Participant 33*

Other providers noted the group basis of DBT-SG would have the advantage of extending services to more veterans at once than a comprehensive DBT program:

*“I know that before I was at this VA, they had actually a DBT program that was very well respected. I think, honestly, with the amount of people that needed that treatment, it wasn’t as efficient at getting to all of them with all of the manpower that went into it. So, it ended up being discontinued. I think that this attempt to offer the same model in a group approach where it could be disseminated amongst a lot more veterans at one time is a great idea.” – Participant 3*

Another provider noted there was high need for DBT at their site, but limited therapists who could provide individual DBT, making DBT-SG an attractive option:

*“One of the benefits of having just skills group is that we don’t have enough providers to offer full DBT to everybody. And so that has helped us meet the need of folks who would benefit from DBT, but without us having to have more individual therapists available to provide it.” – Participant 25*

Providers highlighted DBT-SG’s potential benefit to veterans who are at chronic suicide risk, are impulsive, or have limited coping skills. One provider noted that they liked the broad applicability of DBT-SG for veterans:

*“I think that DBT has a very wide application, which is one of the reasons that I really like it. It works really well with veterans who have a history of complex trauma or just trauma in general.” – Participant 20*

Providers were generally in support of delivering DBT-SG using telehealth and hybrid approaches, although there were some concerns about poor internet connections, technological barriers, and distractions while participating from home. However, respondents concurred that offering DBT-SG was important whether in person or virtually. As one supervisor said,

*“I’m an old-fashioned guy who prefers face-to-face. But I will try to go with the times. The most important thing is to get people in the boat. Everybody has the ability to work virtually” – Participant 16*

##### Recipients

At each of the four sites, interviewees explored unique goals, skills, knowledge, resources, and support that may be most beneficial to the veterans at their facility. Interviewees noted awareness that the cultural influences on veterans would need to shape how DBT-SG was delivered. One provider commented,

*“We have a lot of first- and second-generation Americans in our group so we need to be more culturally sensitive about things.” – Participant 4*

Age of veterans, racial diversity, time since military separation, homelessness, and gender were also identified as variables that could influence the success of DBT-SG. In terms of which providers should deliver DBT-SG at VHA, training in a specific mental health discipline was seen as less important than having adequate DBT-SG training, strong emotion regulation skills, compassion, and the ability to be firm. One provider noted that the most important aspect was ‘strong therapists’:

*“You have to have strong therapists with DBT. The more engaging and invested the therapist is with the therapy … patients pick up on that. And that makes a big difference.” – Participant 20*

##### Inner context

Respondents identified several local organizational factors that could potentially hinder or aid in DBT-SG implementation. Barriers included staffing limitations, provider burnout, and for some respondents, a perception of limited support from local leadership. One provider noted:

*“The lack of manpower is the greatest barrier. There are a lot of people who want to be in the groups as well as (providers) who want to run the groups, but their responsibilities are great and they may not have time.” – Participant 5*

Another provider shared:

*“Barriers would definitely be staffing. And to some extent, burnout. Because we know that this population has a lot of needs and can require a lot of time.” – Participant 8*

Many providers shared that the leadership at their local sites were very supportive of DBT-SG as a suicide prevention intervention. As one provider said:

*“Mental health leadership would be incredibly supportive of an outpatient DBT skills group at our site. We have a special interest group that focuses on suicide prevention specifically and higher up leadership within our local VA very much treat prevention as a priority.” – Participant 21*

However, some providers noted that local leadership were less supportive of group-delivered or intensive individual psychotherapy, describing the past challenges in getting a DBT skills group running:

*“It was very, very, very difficult to get my leadership on board. Our specialty mental health outpatient clinic and our general clinic is run by psychiatry. Psychiatry does not so much see the value of group therapy, and they don’t really see the value of intensive individual therapy. Their interest is really only in getting uniques. Like having therapy providers assess as many new people as we can. That’s how they get their bonuses, unfortunately. They don’t like intensive programs in this clinic because that doesn’t help them for their purposes. I had to advocate for years to finally get the time in my schedule to offer DBT Skills Group.” – Participant 24*

At another site, a psychologist noted that local leadership had first questioned, but ultimately supported, a longer-duration group therapy serving high-risk veterans:

*“I proposed in [month] a 20 session CBT skills group here, and leadership was supportive. They knew that a lot of the demographic that would be enrolled in this group would be our high-risk vets. The idea is that, based on also the literature you’re describing, if we can at least get them enrolled in these skills groups and get them the foundational skills, they may not need to come back for additional episodes of care. That may actually be sufficient. So, they [leadership] had questions about why 20 sessions, but they ultimately were like, yes, this would be a good thing.” –Participant 20*

Several providers who indicated their leadership would be supportive of DBT-SG expressed that leadership support may not be sufficient to overcome limited staffing:

*“I think they’re [leadership are] supportive generally. It would just depend on the level of staffing available to kind of cover any gaps that may come from a provider being pulled away from a certain clinic in order to make this one work.” – Participant 11*

*“Yes, leadership and management would be very on board to provide any assistance or opportunity to say, “if you in clinic can manage this, I’m on board,” but it’s going to come down to individual clinics saying we have the caseload that we can allow admin time for this. And right now, I’m not sure how many of our local clinics have that opportunity.” – Participant 14*

##### Outer context

Both local and national policy drivers and priorities were discussed as having a potential impact on DBT-SG implementation. Providers spoke about suicide prevention as a priority for leadership, resulting in local and national leadership support for programs such as DBT-SG. As one local administrator said:

*“From a systems perspective if this decreases suicide ideation, the implication of that is that it will likely be integrated and added as a part of a practice guideline. We have to prioritize this if it saves lives, it’s shifting the framework and conceptualization of it. With financial support for training, I think the VA messaging would have to be very clear that we’re supporting this as an initiative.” – Participant 26*

A national leader spoke of the benefits of having groups such as DBT implemented for suicide prevention in VHA:

*“A skills group is kind of a low hanging fruit for a facility to implement. You might not catch everyone that you would like to take advantage of that modality, but I think there will be enough people that could fill the seats and maybe go the distance.” – Participant 22*

### Mixed-methods

**Table 4** shows the comparison and integration of the quantitative results and qualitative findings. Qualitative findings supported the quantitative results for all three domains of the i-PARIHS framework examined. In the evidence for the innovation subscale, average ORCA scores indicating agreement that evidence from RCTs, clinical experiences, and patient preferences supported the DBT-SG intervention converged with qualitative findings that DBT-SG is helpful and widely applicable for veterans across the VHA system. Both inner and outer context domains had lower ORCA scores, indicating neutral to slight agreement, which converged with qualitative findings that noted potential key barriers to DBT-SG implementation including staffing and staff burnout. Conversely, important facilitators included leadership support and institutional prioritization of suicide prevention.

**Table 4:** Comparison and Integration of Quantitative and Qualitative Findings.

i-PARIHS Domain	Quantitative Data	Qualitative Data
Evidence for the Innovation	Q: Is DBT-SG innovation supported by evidence?	Q: What elements of the DBT-SG innovation will be helpful to high-risk veterans?
	A: Yes, including evidence from randomized controlled trials (mean ± SD: 4.1 ± 0.7), clinical experiences (mean ± SD: 4.3 ± 0.6), and patient preferences (mean ± SD: 4.1 ± 0.5). No differences noted by study role (referring provider or study therapist vs. other; **Table 3A**) or by leadership status (leadership role vs. non-leadership role; **Table 3B**).	A: DBT skills can be helpful even without DBT individual therapy or phone coaching; DBT skills have a wide application, including for veterans with trauma; staff in support of DBT-SG being provided for a wide variety of Veterans
Inner context	Q: Do general resources support DBT-SG at VHA?	Q: In what ways might general resources support or impede DBT-SG implementation at VHA?
	A: Neutral to slightly agree or slightly disagree (mean ± SD: 3.3 ± 0.9). Referring providers and study therapists had lower confidence in resources and staff culture supporting DBT-SG implementation than those in other roles (**Table 3A**), while those in local leadership positions had higher confidence in resources than those in non-leadership roles (**Table 3B**).	A: Lack of staffing is a key barrier to DBT-SG implementation; some concern that provider burnout may also be a barrier. Although local leadership is perceived as supportive and staff are eager to provide DBT-SG, low staffing could limit implementation.
Outer context	Q: Do leadership behavior, feedback, and culture support DBT-SG implementation at VHA?	Q: In what ways might leadership and policy support DBT-SG implementation at VHA?
	A: Neutral to slightly agree for all: leadership behavior (mean ± SD: 3.5 ± 1.1), feedback from leadership (mean ± SD: 3.5 ± 1.0), and leader culture (mean ± SD: 3.8 ± 1.0). Referring providers and study therapists had lower confidence in leadership behavior, feedback, and culture (**Table 3A**), while those in local leadership positions had higher confidence in each domain than those in non-leadership roles (**Table 3B**).	A: Suicide prevention is widely perceived as a high institutional priority; staff anticipate DBT-SG being integrated as part of the overall suicide prevention initiative at VHA systems

The Organizational Readiness to Change Assessment (ORCA; Helfrich, Sharp, & Sales, 2009) included items from the three domains of the original Promoting Action on Research in Health Services (PARIHS; Kitson, Harvey, & McCormack, 1998) assessing the strength of evidence for the innovation, quality of the context for implementation, and capacity to facilitate the implementation. This study used the ORCA evidence and context subscales. The revised Integrated Promoting Action on Research in Health Services (i-PARIHS; Harvey & Kitson, 2016) added the construct of recipients, which is not formally included in the ORCA scales used in this study. All quantitative items are scored as 1=Strongly Disagree | 2=Disagree | 3=Neither Agree nor Disagree | 4=Agree | 5=Strongly Agree.

## Discussion

This mixed-method formative evaluation of implementation determinants to DBT-SG at four VHA sites found that quantitative and qualitative data both showed VHA staff had favorable views of the innovation overall. Furthermore, both quantitative and qualitative data converged to elucidate staff views about inner context barriers (i.e., site-level), such as provider shortage and concern for local resources. Qualitative data highlighted potential facilitators, such as local leadership support for implementing DBT-SG, and national policy drivers that prioritized suicide prevention interventions for high-risk veterans.

ORCA scores showed VHA staff agreed that DBT-SG was supported by evidence from research, patient preferences, and clinical experience. Qualitative data expanded on this to show VHA staff’s interest in suicide prevention interventions that can be transdiagnostic, consistent with reviews showing DBT skills interventions have been studied well beyond individuals with BPD (27). Previous clinical practice guidelines for VHA offered support for DBT for veterans at risk for suicide who have BPD (45), and the current Suicide Prevention 2.0 initiative extends DBT to veterans with history of suicidal self-directed violence who have BPD (16). While conserving resource-intensive treatments like comprehensive DBT for those who are most likely to benefit based on similarity to research findings is reasonable, our data suggest VHA staff see transdiagnostic utility in the DBT-SG intervention for veterans at risk for suicidal behavior, beyond the BPD diagnosis.

Qualitative data showed VHA staff awareness of the wide range of diversity that might influence how veterans interact with DBT-SG concepts. United States ethno-racial and sexual orientation minority groups are well represented in comprehensive DBT randomized controlled trials, supporting the use of comprehensive DBT for individuals from a wide range of demographic identities (46). To ensure DBT-SG at VHA is delivered with attention to veteran diversity, we recommend that DBT-SG providers in VHA use therapist consultation team to support delivering treatment with cultural competence and to ensure effective treatment of veterans who experience racism and other forms of minority stress (47). Regarding recommended DBT-SG therapist characteristics, our data are consistent with other DBT implementation literature emphasizing the importance of therapist qualities such as cognitive flexibility or open-mindedness (23, 30), assertiveness or interpersonal effectiveness (23, 30, 48), therapist interest or investment in the treatment model (18, 21, 23), and adequate training in the treatment (21, 23, 30).

Within each medical center (i.e., inner context), quantitative data (**Table 2**) showed a belief that VHA staff have a personal commitment to improving veteran care and outcomes, which combined with staff perception of DBT-SG as having strong evidence as a suicide prevention treatment based on clinical trials, clinical experience, and patient preference, bodes well for implementing DBT-SG in VHA medical centers (49). However, there was less confidence in the adequacy of resources or leadership behavior to support DBT-SG implementation. This pattern of higher ORCA mean scores for the evidence of an innovation relative to contextual leadership support for implementing it is similar to prior studies using the ORCA within three quality improvement projects conducted within VHA medical settings (37), VHA addiction treatment programs adopting hepatitis prevention practices (49), and academic medical centers promoting medications for alcohol use disorder (50) and buprenorphine (51). Qualitative data showed that both providers and local administrators were invested in providing DBT-SG, but aware that limited staffing could be an insurmountable barrier. Unfortunately, high workload and insufficient staffing have been barriers to previous VHA provision of DBT (21) and other evidence-based psychotherapies (52, 53).

In examining quantitative responses by roles, no differences emerged for the evidence support for DBT-SG based on whether the respondent was a study therapist or referring provider, or had a different role (**Table 3A**), or whether the respondent held a leadership role at the local VHA medical center (**Table 3B**). However, study therapists and referring providers, who would have the most direct relationship to providing or recommending DBT-SG, showed lower confidence that the organizational context (leadership culture, staff culture, leadership behavior, feedback from leadership, and resources) would support DBT-SG implementation, in comparison to respondents in leadership roles (**Table 3A**). Similar results on the resources subscale emerged in another pre-implementation study: prescribers who would directly prescribe medication for alcohol use disorder in an inpatient medical setting showed lower resource subscale scores (*M* = 2.8, 95% CI (2.5, 3.2), *n* = 26) than non-prescribers (*M* = 3.3, 95% CI (3.0, 3.6), *n* = 27 (50).

In our data, holding a leadership position at the local facility also differentiated some results in the ORCA context subscales: individuals who were in a leadership role (hospital director, administrative leader, clinical supervisor) showed greater confidence in leadership culture, leadership behavior, feedback from leadership, and resources (**Table 3B**), suggesting that leaders had a more positive perception of the implementation context for DBT-SG than those without leadership responsibilities, despite similar views on the evidence for DBT-SG. It is unclear which perception is more accurate. DBT clinicians have previously pointed out that organizational support is critical to implementation success (20, 21). In a prior VHA DBT program evaluation (21), DBT team leaders or point of contact identified barriers their site was working on or could not overcome included lack of individual therapists, lack of therapist availability to take on enough patients, inability to schedule a two-hour group, and inability to block time for consultation team. Similarly, in a large evaluation of DBT program sustainability in the UK, absence of organizational support was the most commonly identified implementation barrier, while presence of organizational support was the most commonly identified implementation facilitator (20).

Our data and prior literature suggest that implementing DBT-SG at trial sites may benefit from an implementation strategy that emphasizes the perspectives of DBT-SG clinicians and referring providers, such as implementation facilitation (31), a dynamic implementation strategy that includes problem-solving, enabling, and supporting implementation partners or stakeholders as they implement a clinical innovation into routine practice (54). Core implementation facilitation activities emphasize engaging stakeholders or partners to evaluate context, resources, and barriers, and then assisting them in tailoring or adapting a clinical innovation to their setting (55). Implementation facilitation requires targeting all groups and stakeholders who can impact implementation, including clinical providers who will implement the innovation, providers and staff who would refer to the innovation, and organizational leaders who could support, or inadvertently impede, implementation efforts (56).

Regarding outer context, in qualitative data VHA providers emphasized that their leaders are invested in suicide prevention, suggesting the outer context of policy and initiatives focused on suicide prevention (2) is evident to providers. However, VHA staff noted awareness that national leadership need to devote adequate resources to DBT-SG: as one local administrator said, “we have to prioritize this if it saves lives.” Across VHA, mental health clinical provider staffing levels were causally linked to lower probability of veteran suicide-related events (57), suggesting the need to increase staffing levels or improve the efficiency of currently available providers. In fiscal year 2024, VHA facilities surveyed identified severe staffing shortage in clinical mental health occupations including psychology (identified as a severe shortage by 61% facilities), psychiatry (47%), and social work (43%; 58). To further prioritize veteran suicide prevention, national leadership may need to consider prioritizing funding to support greater clinical VHA mental health provider staffing (57).

Strengths of this investigation include use of an established implementation science framework to guide study interview development and survey selection, mixed methods to examine the complementarity and expansion of data and fully capture staff views, data gathering across four sites and different categories of staff, and sampling to saturation for qualitative interviews. Limitations, as with other mixed method studies, include that these findings are not necessarily expected to generalize to other settings. Veteran experiences and views were not captured in these data and will be included in subsequent formative evaluation during the ongoing hybrid effectiveness-implementation trial.

Further mixed methods directions include qualitative interviews with veterans at high-risk for suicide attempt who participated in DBT-SG, examining the experiences of those who completed the treatment and those who dropped out (59, 60), using purposeful sampling to understand variation in participant experience (61). We will also gather interview- and ORCA-derived data about DBT-SG therapist, referring provider, and leadership perspectives on how to best facilitate the implementation of DBT-SG based on their views of the use of DBT-SG during our trial. This information will inform development of an implementation strategy for DBT-SG, should the treatment demonstrate effectiveness in improving emotion regulation of veterans and reducing their rates of suicide attempt.

## Data Availability

The datasets presented in this article are not readily available because, in consultation with Institutional Review Board, data sets are not shared due to the sensitive topic. Requests to access the datasets should be directed to suzanne.decker@va.gov.

## References

[1] Department of Veterans Affairs. National veteran suicide prevention annual report (2023). Available online at: https://www.mentalhealth.va.gov/docs/data-sheets/2023/2023-National-Veteran-Suicide-Prevention-Annual-Report-FINAL-508.pdf (Accessed September 9, 2024).

[2] White House. Reducing military and veteran suicide: Advancing a comprehensive, cross-sector, evidence-informed public health strategy (2021). Available online at: https://www.whitehouse.gov/wp-content/uploads/2021/11/Military-and-Veteran-Suicide-Prevention-Strategy.pdf (Accessed May 2, 2024).

[3] U.S. Department of Veterans Affairs. National strategy for preventing Veteran suicide, 2018-2028 (2018). Office of Mental Health and Suicide Prevention. Available online at: https://www.mentalhealth.va.gov/suicide_prevention/docs/Office-of-Mental-Health-and-Suicide-Prevention-National-Strategy-for-Preventing-Veterans-Suicide.pdf (Accessed September 9. 2024).

[4] Ilgen MA Bohnert AS Ignacio RV McCarthy JF Valenstein MM Kim HM. Psychiatric diagnoses and risk of suicide in veterans. Arch Gen Psychiatry. (2010) 67:1152–8. doi: 10.1001/archgenpsychiatry.2010.129 21041616

[5] Sloan E Hall K Moulding R Bryce S Mildred H Staiger PK. Emotion regulation as a transdiagnostic treatment construct across anxiety, depression, substance, eating and borderline personality disorders: A systematic review. Clin Psychol Rev. (2017) 57:141–63. doi: 10.1016/j.cpr.2017.09.002 28941927

[6] Hazlett EA Blair NJ Fernandez N Mascitelli K Perez-Rodriguez MM New AS. Startle amplitude during unpleasant pictures is greater in veterans with a history of multiple-suicide attempts and predicts a future suicide attempt. Psychophysiology. (2016) 53:1524–34. doi: 10.1111/psyp.12698 27378071

[7] Denning DM Perry TR Reilly EE Berner LA Velkoff EA Kaye WH. Associations of suicide risk with emotional reactivity, dysregulation, and eating disorder treatment outcomes. Suicide Life Threat Behav. (2022) 53:1126–39. doi: 10.1111/sltb.12907 PMC10394756 36082588

[8] Rufino KA Ward-Ciesielski EF Webb CA Nadorff MR. Emotion regulation difficulties are associated with nightmares and suicide attempts in an adult psychiatric inpatient sample. Psychiatry Res. (2020) 293:113437. doi: 10.1016/j.psychres.2020.113437 32916439

[9] Rudd MD Williams B Trotter DRM. The psychological and behavioral treatment of suicidal behavior: What are the common elements of treatments that work? In: Wasserman D Wasserman C, editors. Oxford textbook of suicidology and suicide prevention. Oxford University Press, Oxford, United Kingdom (2008). p. 427–38.

[10] Linehan MM. Cognitive-behavior therapy for borderline personality disorder. New York: Guilford Press (1993).

[11] DeCou CR Comtois KA Landes SJ. Dialectical behavior therapy is effective for the treatment of suicidal behavior: A meta-analysis. Behav Ther. (2019) 50:60–72. doi: 10.1016/j.beth.2018.03.009 30661567

[12] Gratz KL Lacroce DM Gunderson JG. Measuring changes in symptoms relevant to borderline personality disorder following short-term treatment across partial hospital and intensive outpatient levels of care. J Psychiatr Pract. (2006) .2:153–9. doi: 10.1097/00131746-200605000-00004 16732134

[13] Pistorello J Fruzzetti AE Maclane C Gallop R Iverson KM. Dialectical behavior therapy (DBT) applied to college students: a randomized clinical trial. J Consult Clin Psychol. (2012) 80:982–94. doi: 10.1037/a0029096 PMC3514574 22730955

[14] Goldstein TR Merranko J Rode N Sylvester R Hotkowski N Fersch-Podrat R. Dialectical Behavior Therapy for adolescents with bipolar disorder: A randomized clinical trial. JAMA Psychiatry. (2024) 81:15–24. doi: 10.1001/jamapsychiatry.2023.3399 37703037 PMC10500432

[15] Goodman M Banthin D Blair NJ Mascitelli KA Wilsnack J Chen J. A randomized trial of dialectical behavior therapy in high-risk suicidal veterans. J Clin Psychiatry. (2016) 77:e1591–e600. doi: 10.4088/JCP.15m10235 27780335

[16] Walker J Betthauser LM Green K Landes SJ Stacy M. Suicide Prevention 2.0 Clinical Telehealth Program: Evidence-Based Treatment in the Veterans Health Administration. 2024 April 28; virtual (2024). Available online at: https://www.youtube.com/watch?v=fFsDzkg0SR0. (Accessed September 17, 2024).

[17] Comtois KA Landes SJ. Implementing DBT: An implementation science perspective. In: Swales MA, editor. The oxford handbook of dialectical behaviour therapy. Oxford University Press, Oxford, United Kingdom (2018). p. 831–44.

[18] Herschell AD Kogan JN Celedonia KL Gavin JG Stein BD. Understanding community mental health administrators’ perspectives on dialectical behavior therapy implementation. Psychiatr Serv. (2009) 60:989–92. doi: 10.1176/ps.2009.60.7.989 PMC5317039 19564234

[19] Carmel A Rose ML Fruzzetti AE. Barriers and solutions to implementing dialectical behavior therapy in a public behavioral health system. Adm Policy Ment Health. (2014) 41:608–14. doi: 10.1007/s10488-013-0504-6 PMC3835762 23754686

[20] Swales MA Taylor B Hibbs RA. Implementing Dialectical Behaviour Therapy: programme survival in routine healthcare settings. J Ment Health. (2012) 21:548–55. doi: 10.3109/09638237.2012.689435 22958107

[21] Landes SJ Rodriguez AL Smith BN Matthieu MM Trent LR Kemp J. Barriers, facilitators, and benefits of implementation of dialectical behavior therapy in routine care: results from a national program evaluation survey in the Veterans Health Administration. Transl Behav Med. (2017) 7:832–44. doi: 10.1007/s13142-017-0465-5 PMC5684063 28168608

[22] King JC Hibbs R Saville CWN Swales MA. The survivability of dialectical behaviour therapy programmes: Amixed methods analysis of barriers and facilitators to implementation within UK healthcare settings. BMC Psychiatry. (2018) 18:302. doi: 10.1186/s12888-018-1876-7 30231865 PMC6146662

[23] Quetsch LB Herschell AD Kogan JN Gavin JG Hale G Stein BD. Community-based behavioral health administrator perspectives on sustainability of Dialectical Behavior Therapy: A qualitative evaluation. Borderline Pers Disord Emot Dysregul. (2020) 7:5. doi: 10.1186/s40479-020-0120-5 PMC7047370 32161650

[24] Linehan MM Korslund KE Harned MS Gallop RJ Lungu A Neacsiu AD. Dialectical behavior therapy for high suicide risk in individuals with borderline personality disorder: A randomized clinical trial and component analysis. JAMA Psychiatry. (2015) 72:475–82. doi: 10.1001/jamapsychiatry.2014.3039 25806661

[25] McMain SF Guimond T Barnhart R Habinski L Streiner DL. A randomized trial of brief dialectical behaviour therapy skills training in suicidal patients suffering from borderline disorder. Acta Psychiatrica Scandinavica. (2017) 135:138–48. doi: 10.1111/acps.12664 27858962

[26] Neacsiu AD Eberle JW Kramer R Wiesmann T Linehan MM. Dialectical behavior therapy skills for transdiagnostic emotion dysregulation: A pilot randomized controlled trial. Beha Res Ther. (2014) 59:40–51. doi: 10.1016/j.brat.2014.05.005 24974307

[27] Valentine SE Bankoff SM Poulin RM Reidler EB Pantalone DW. The use of dialectical behavior therapy skills training as stand-alone treatment: a systematic review of the treatment outcome literature. J Clin Psychol. (2015) 71:1–20. doi: 10.1002/jclp.22114 25042066

[28] Larkin C Arensman E Boudreaux ED. Preventing suicide in health systems: How can implementation science help? Arch Suicide Res. (2023) 27:1147–62. doi: 10.1080/13811118.2022.2131490 36267036

[29] Allison MK Waliski A Haynes TF Marshall SA. Formative evaluation of Zero Suicide in the emergency department: Identifying strategies to overcome implementation barriers. Eval Program Plann. (2022) 92:102050. doi: 10.1016/j.evalprogplan.2022.102050 35217479

[30] Toms G Williams L Rycroft-Malone J Swales M Feigenbaum J. The development and theoretical application of an implementation framework for dialectical behaviour therapy: a critical literature review. Borderline Pers Disord Emot Dysregul. (2019) 6:2. doi: 10.1186/s40479-019-0102-7 PMC6373034 30805193

[31] Harvey G Kitson A. PARIHS revisited: from heuristic to integrated framework for the successful implementation of knowledge into practice. Implement Sci. (2016) 11:33. doi: 10.1186/s13012-016-0398-2 27013464 PMC4807546

[32] Curran GM Bauer M Mittman B Pyne JM Stetler C. Effectiveness-implementation hybrid designs: combining elements of clinical effectiveness and implementation research to enhance public health impact. Med Care. (2012) 50:217–26. doi: 10.1097/MLR.0b013e3182408812 PMC3731143 22310560

[33] Landes SJ McBain SA Curran GM. An introduction to effectiveness-implementation hybrid designs. Psychiatry Res. (2019) 280:112513. doi: 10.1016/j.psychres.2019.112513 31434011 PMC6779135

[34] Palinkas LA Aarons GA Horwitz S Chamberlain P Hurlburt M Landsverk J. Mixed method designs in implementation research. Adm Policy Ment Health. (2011) 38:44–53. doi: 10.1007/s10488-010-0314-z 20967495 PMC3025112

[35] Stetler CB Legro MW Wallace CM Bowman C Guihan M Hagedorn H. The role of formative evaluation in implementation research and the QUERI experience. J Gen Intern Med. (2006) 21 Suppl 2:S1–8. doi: 10.1111/j.1525-1497.2006.00355.x PMC2557128 16637954

[36] Harris PA Taylor R Thielke R Payne J Gonzalez N Conde JG. Research electronic data capture (REDCap)–a metadata-driven methodology and workflow process for providing translational research informatics support. J BioMed Inform. (2009) 42:377–81. doi: 10.1016/j.jbi.2008.08.010 PMC2700030 18929686

[37] Helfrich CD Li YF Sharp ND Sales AE. Organizational readiness to change assessment (ORCA): development of an instrument based on the Promoting Action on Research in Health Services (PARIHS) framework. Implement Sci. (2009) 4:38. doi: 10.1186/1748-5908-4-38 19594942 PMC2716295

[38] Mattocks K Rosen MI Sellinger J Ngo T Brummett B Higgins DM. Pain care in the Department of Veterans Affairs: Understanding how a cultural shift in pain care impacts provider decisions and collaboration. Pain Med. (2020) 21:970–7. doi: 10.1093/pm/pnz341 PMC7208326 31886869

[39] Hamilton A. Qualitative methods in rapid turn-around health services research. VA HSR&D. https://www.hsrd.research.va.gov/for_researchers/cyber_seminars/archives/780-notes.pdf. (Accessed September 01, 2024).

[40] Hamilton AB Finley EP. Qualitative methods in implementation research: An introduction. Psychiatry Res. (2019) 280:112516. doi: 10.1016/j.psychres.2019.112516 31437661 PMC7023962

[41] Gabrielian S Finley EP Ganz DA Barnard JM Jackson NJ Montgomery AE. Comparing two implementation strategies for implementing and sustaining a case management practice serving homeless-experienced veterans: A protocol for a type 3 hybrid cluster-randomized trial. Implement Sci. (2022) 17:67. doi: 10.1186/s13012-022-01236-1 36192785 PMC9527738

[42] Averill JB. Matrix analysis as a complementary analytic strategy in qualitative inquiry. Qual Health Res. (2002) 12:855–66. doi: 10.1177/104973230201200611 12109729

[43] Fereday J Muir-Cochrane E. Demonstrating rigor using thematic analysis: A hybrid approach of inductive and deductive coding and theme development. Intl J Qual Meth. (2006) 5:80–92. doi: 10.1177/160940690600500107

[44] Ritchie J Spencer L. Qualitative data analysis for applied policy research. In: Bryman A Burgess R, editors. Analysing qualitative data. Routledge, London (1993). p. 173–94.

[45] Department of Veterans Affairs, Department of Defense. VA/DoD clinical practice guidelines for assessment and management of patients at risk for suicide (2013). Available online at: https://www.healthquality.va.gov/guidelines/MH/srb/VADoDSuicideRiskFullCPGFinal5088212019.pdf (Accessed September 9. 2024).

[46] Harned MS Coyle TN Garcia NM. The inclusion of ethnoracial, sexual, and gender minority groups in randomized controlled trials of Dialectical Behavior Therapy: A systematic review of the literature. Clin Psychol: Sci Pract. (2022) 29:83–93. doi: 10.1037/cps0000059

[47] Pierson AM Arunagiri V Bond DM. You didn’t cause racism, and you have to solve it anyways”: Antiracist adaptations to Dialectical Behavior Therapy for white therapists. Cog Behav Pract. (2022) 29:796–815. doi: 10.1016/j.cbpra.2021.11.001

[48] Ditty MS Landes SJ Doyle A Beidas RS. It Takes a Village: A mixed method analysis of inner setting variables and Dialectical Behavior Therapy implementation. Adm Policy Ment Health. (2015) 42:672–81. doi: 10.1007/s10488-014-0602-0 PMC4400206 25315183

[49] Hagedorn HJ Heideman P. The relationship between baseline Organizational Readiness to Change Assessment subscale scores and implementation of hepatitis prevention services in substance use disorders treatment clinics: a case study. Implementation Sci. (2010) 5:46. doi: 10.1186/1748-5908-5-46 PMC2902416 20546584

[50] Joudrey PJ Oldfield BJ Yonkers KA O’Connor PG Berland G Edelman JE. Inpatient adoption of medications for alcohol use disorder: A mixed methods formative evaluation involving key stakeholders. Drug Alcohol Dependence. (2020) 213:108090. doi: 10.1016/j.drugalcdep.2020.108090 32559667 PMC7375447

[51] Hawk KF D’Onofrio G Chawarski MC O’Connor PG Cowan E Lyons MS. Barriers and facilitators to clinician readiness to provide emergency department-initiated buprenorphine. JAMA Network Open. (2020) 3:e204561. doi: 10.1001/jamanetworkopen.2020.4561 32391893 PMC7215257

[52] Chard KM Ricksecker EG Healy ET Karlin BE Resick PA. Dissemination and experience with cognitive processing therapy. J Rehabil Res Dev. (2012) 49:667–78. doi: 10.1682/jrrd.2011.10.0198 23015578

[53] Finley EP Garcia HA Ketchum NS McGeary DD McGeary CA Stirman SW. Utilization of evidence-based psychotherapies in Veterans Affairs posttraumatic stress disorder outpatient clinics. Psychol Serv. (2015) 12:73–82. doi: 10.1037/ser0000014 25419915 PMC4333008

[54] Powell BJ Waltz TJ Chinman MJ Damschroder LJ Smith JL Matthieu MM. A refined compilation of implementation strategies: Results from the Expert Recommendations for Implementing Change (ERIC) project. Implementation Sci. (2015) 10:21. doi: 10.1186/s13012-015-0209-155 PMC4328074 25889199

[55] Smith JL Ritchie MJ Kim B Miller CJ Chinman MJ Kelly A. Getting to fidelity: Consensus development process to identify core activities of implementation facilitation. Global Implementation Res Applications. (2024) 4:151–66. doi: 10.1007/s43477-024-00119-5 PMC11100021 38765294

[56] Ritchie MJ Dollar KM Miller CJ Smith JL Oliver KA Kim B. Using implementation facilitation to improve healthcare (Version 3). North Little Rock, AR: Veterans Health Administration, Behavioral Health Quality Enhancement Research Initiative (QUERI). (2020). Available at: https://www.queri.research.va.gov/tools/Facilitation-Manual.pdf.

[57] Feyman Y Figueroa SM Yuan Y Price ME Kabdiyeva A Nebeker JR. Effect of mental health staffing inputs on suicide-related events. Health Serv Res. (2023) 58:375–82. doi: 10.1111/1475-6773.14064 PMC10012216 36089760

[58] U.S. Department of Veterans Affairs Office of Inspector General. OIG determination of veterans health administration’s severe occupational staffing shortages - fiscal year 2024. 2024 August 7, 2024. Report No.: 24-00803-222 . Available online at: https://www.vaoig.gov/reports/national-healthcare-review/oig-determination-veterans-health-administrations-severe-0 (Accessed October 11, 2024).

[59] Patel SR Sullivan SR Mitchell EL Jager-Hyman S Stanley B Goodman M. Qualitative study of telehealth delivery of suicide-specific group treatment “Project Life Force. J Technol Behav Sci. (2023) 8:272–81. doi: 10.1007/s41347-022-00297-9 PMC9811055 36618084

[60] Barnicot K Couldrey L Sandhu S Priebe S. Overcoming barriers to skills training in Borderline Personality Disorder: A qualitative interview study. PloS One. (2015) 10:e0140635. doi: 10.1371/journal.pone.0140635 26465757 PMC4605586

[61] Palinkas LA Horwitz SM Green CA Wisdom JP Duan N Hoagwood K. Purposeful sampling for qualitative data collection and analysis in mixed method implementation research. Adm Policy Ment Health. (2015) 42:533–44. doi: 10.1007/s10488-013-0528-y PMC4012002 24193818

